# Ethical considerations for researchers developing and testing minimal-risk devices

**DOI:** 10.1038/s41467-023-38068-6

**Published:** 2023-04-22

**Authors:** Anna Wexler, Emily Largent

**Affiliations:** Department of Medical Ethics & Health Policy, Perelman School of Medicine, University of Pennsylvania, Philadelphia, PA USA

**Keywords:** Ethics, Policy, Institutions, Engineering, Materials for devices

## Abstract

This comment explores ethical aspects in developing and testing minimal-risk devices, such as wearables and biomedical sensors. Authors outline the process of independent review, emphasizing the different levels of review depending on the research design and risk level. They also share examples of practical scenarios, highlighting key ethical considerations.

## Teaser

This comment provides an overview of ethical considerations for researchers developing and testing minimal-risk devices that interact or interface with people, such as electronic wearables and biomedical sensors. We outline the process of independent review, emphasizing that research can undergo different levels of review depending on its design and risk level. Using scenarios drawn from our own experience, we outline salient ethical considerations for research with such minimal-risk devices.

An increasing number of tools and devices that directly interact or interface with people for electronic wearable and bioengineering applications are being developed, tested, and marketed to consumers^1^. For example, wearable devices, which may record everything from skin temperature to electrical brainwave signals, enable wearers to monitor a wide range of information generated by their bodies. Part of the device development process includes prototyping and testing, as researchers must establish the reliability and validity of their wearable by having people use it. Many engineers and materials scientists may, however, not be aware that they should obtain approval from an independent review committee prior to testing their device with people. This independent oversight requirement does not merely apply to clinical trials testing novel drugs or devices in patient populations; it can also extend to researchers and engineers developing seemingly “harmless” sensors or conducting “quick” validation tests on themselves or members of their lab group.

In the process known as “independent review,” researchers submit a detailed study plan or “protocol” to a group of experts not affiliated with the research who are able to assess the protocol and ensure its compliance with ethical standards and relevant research regulations. Independent review can be a legal requirement (e.g., for government-funded research or research submitted to drug or device regulators) or an institutional one (e.g., if the research is conducted under the auspices of a university or academic medical center). Other gatekeepers may also require independent review. For example, before publication, many academic journals require authors to affirm their studies were conducted with adequate participant protections, and platforms such as Apple’s app store require evidence of independent ethics review before distributing software that collects data for research purposes^2^. Notably, compliance with such requirements cannot be achieved retrospectively; thus, researchers must address independent review prospectively or risk myriad consequences.

In this article, we provide an overview of research ethics considerations for researchers developing and testing wearable health devices with human subjects. We first review the importance of independent review. Next, we outline the process of independent review, emphasizing that research can undergo different levels of review depending on its design and risk level. Finally, using scenarios drawn from our real-word experience, we outline salient ethical considerations for research with wearables and describe the steps researchers can take to ensure compliance with ethics requirements.

## Why is independent review important?

Though a researcher may simply be seeking to better understand the performance or function of their wearable device, if they are doing so by interacting with and collecting information from living individuals, they are conducting “human subjects research” (see Table 1 for definition). Multiple national and international consensus documents have set forth ethical guidelines for research conducted with human subjects^3^. There are notable commonalities across these research ethics guidelines, which have been distilled by prominent research ethicists into seven requirements for ethical research^3^. These requirements apply to human subjects research, including but not limited to research with wearable health devices. The research must be (1) socially valuable and (2) scientifically rigorous, as well as offer (3) a favorable risk–benefit ratio. Subjects must (4) be selected fairly and (5) give informed consent for their participation. Researchers must (6) demonstrate respect for potential and enrolled subjects. Finally, the research must (7) undergo independent review. As we discuss below, independent review helps to assure compliance with relevant ethical and regulatory requirements and to address additional issues as they arise.

**Table 1:** Minimal-risk research with human subjects: definitions from the U.S. Common Rule^16^ and examples

Term	Definition	Examples
Research	“systematic investigation…designed to develop… generalizable knowledge”	Student studying the properties of soft materials for thesis; scientist conducting grant-funded clinical trial involving a behavioral intervention; citizen scientist testing a new device and aiming to present findings at an academic conference *Not research:* private company testing a quality-improvement modification to a consumer product
Human subjects research	Research that involves “identifiable private information” or data obtained through “intervention or interaction” with a living individual	Researcher conducting surveys or interviews; researcher testing biomedical sensors in humans; self-experimentation with a heart rate monitor for the purposes of developing generalizable knowledge *Not human subjects research*: scientists utilizing deidentified data or publicly available information
Minimal risk	“the probability and magnitude of harm or discomfort anticipated in the research are not greater in and of themselves than those ordinarily encountered in daily life”	Research utilizing most wearable, noninvasive biomedical sensors; research involving surveys and interviews *Not minimal-risk:* research involving invasive or implantable devices; research involving a pharmaceutical intervention

Researchers have certain reasonable interests—such as the desire to publish, develop and market a new wearable health device, or obtain grant funding—that may not be aligned with subjects’ interests. Even well-intentioned researchers may be unable to reflect impartially on their own research. Review by individuals unaffiliated with the research provides an opportunity for impartial assessment to minimize the effects of any conflicts of interest and ensure appropriate safeguards are in place to protect and promote subjects’ wellbeing^3^.

For this reason, numerous stakeholders might require independent review. For instance, in the United States, independent review is required for clinical investigations of interventions—such as drugs, biologics, and devices—under the jurisdiction of the U.S. Food and Drug Administration, as well as for human subjects research funded by many federal agencies. Institutions engaged in federally-funded human subjects research, such as universities and academic medical centers, must comply with federal research regulations, and many elect to extend independent review requirements to all human subjects research conducted under their auspices, regardless of funding source. Similar regulations and guidelines exist across the globe^3,4^.

Before prototyping and testing their wearable devices, researchers should acquaint themselves with any relevant independent review requirements. This involves becoming familiar with both legal and local institutional requirements. Furthermore, even absent a legal or institutional requirement, independent review may be necessary to satisfy other stakeholders, including private funders, academic journals, or app platforms. Thus, researchers should also consider their funding source as well as how they might want to disseminate their findings or market their wearable health devices and address any oversight requirements entailed by those choices.

## Who conducts independent review, and how does it proceed?

Independent review is undertaken by committees officially constituted to oversee human subjects research^5^. In the United States, these are known as institutional review boards (IRBs); elsewhere they may be known as research ethics committees or independent ethics committees^4–6^. Hereafter, we will use “committee” or “independent review committee” to refer to these bodies. Committee members typically have a range of expertise so that they can provide robust review. Some committees are operated by academic or other institutions, others are operated by governments or funders^7^, and still others are independent (i.e., not affiliated with an institution) and review research for a fee^8^.

While each independent review committee has different requirements, reflecting both their own policies and relevant regulations, a researcher who is conducting human subjects research typically must prepare and submit a detailed protocol that outlines the study objectives, methods, data management and analysis procedures, and plans to address ethical issues that may arise. This protocol is then reviewed by the committee, which can approve it, require modifications to it, or reject it. If a researcher has questions—for instance, regarding the need for independent review or how to prepare a protocol—many committees have websites that provide submission guidelines and resources, as well as contact information for the committee.

**Fig. 1:**
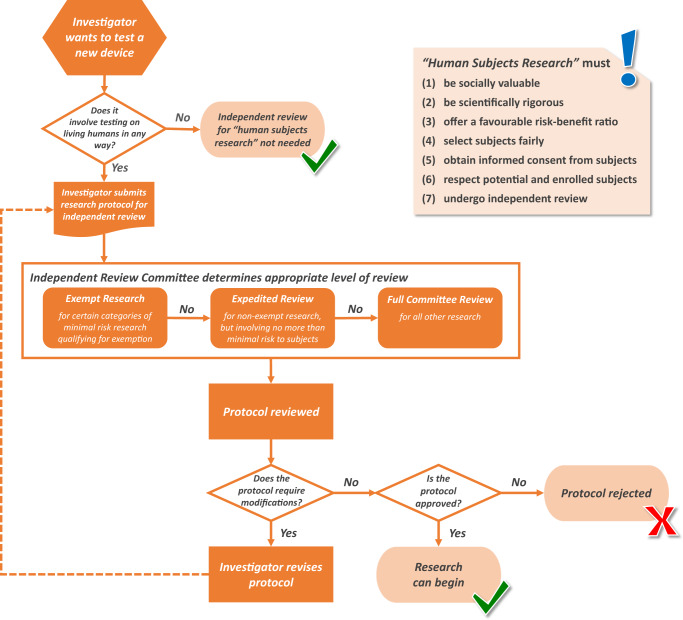
Process for independent review of a device being tested in human subjects in the United States. A researcher developing a new device should carefully consider whether the research must undergo independent review prior to commencing research. If independent review is necessary, the researcher must prepare a protocol and submit it to an independent review committee that will determine the appropriate level of review (i.e., exempt, expedited, or full committee review). The committee will review the protocol, and either approve it, require modifications, or reject it. Research should only begin once the protocol is approved.

Protocols may undergo different levels of review depending on specific aspects of the study’s design and risk profile. For example, in the United States, there are three main designations: *exempt research*, *expedited review*, and *full committee review* (see Fig. 1). Research that involves “minimal risk” (see Table 1) to subjects and includes only behavioral research—such as surveys or passive observation—may be deemed *exempt* from federal regulations and need not undergo IRB review, though the IRB may still need to assess the protocol to confirm its exempt status. Research that involves minimal risk but does not meet the requirements for an exemption may qualify for *expedited review*. For example, data collected through noninvasive means, such as wearable devices and sensors, or via recordings (e.g., audio, textual, or visual) will typically qualify for expedited review. When review is expedited, the protocol may be reviewed by one or more experienced IRB members without the need to convene the full committee, which may result in faster review. All research that is deemed to involve higher levels of risk requires *full committee review*. Importantly, researchers themselves do not determine the appropriate level of review; this assessment is made by the committee after the protocol’s submission.

Independent review committees are generally subject to legislation at the national level, with a goal of better serving local needs and addressing cultural preferences^6^. This can result in substantial variability in review procedures between and within countries and underscores the importance of familiarizing oneself with relevant laws^6,7,9^. While the details do vary, in a broad sense, most committees will look to see that the seven requirements of ethical research, described above, are satisfied.

## What do independent review committees look for in a research protocol involving human subjects?

While it is not possible to cover all ethical considerations relevant to device research, here we use two scenarios of researchers developing wearable health devices to illustrate salient ethical features that researchers should consider. Note that the ethical issues highlighted here are not unique to wearable device research and must also be addressed for other kinds of research with human subjects.

**Scenario 1:** *A university professor is developing a wet adhesive on-skin sensor that assesses both cardiac rhythms and hydration levels. The professor plans to validate the device on students enrolled in a course she teaches. She would like to collect additional data from students’ smartphones, such as location and activity data, to see if she can detect meaningful correlations between physical activity, heart rate, and hydration levels*.

*Fair subject selection*: The proposed sample—students enrolled in the professor’s course—is a “convenience sample,” chosen for being close at hand rather than for the advancement of scientific goals. This raises questions about fair subject selection—that is, whether the subjects are chosen for scientific purposes rather than factors unrelated to the purposes of the study, like vulnerability or privilege. Indeed, this sample may differ from the general population in some way, introducing bias. Thus, in developing study methodology, researchers should ensure that their sample is fairly chosen and justified by the science.

*Risk–benefit ratio*: Although the research described in the above scenario would likely meet the definition of minimal risk, this will be a fact-specific determination, as wet adhesives involve chemicals that can irritate the skin or cause allergic reactions. The committee will consider physical risks as well as psychological, social, and economic risks. The professor in the scenario, like all researchers, should enumerate risks and burdens to subjects, identify and implement steps to minimize them, and—once they are minimized—weigh whether the potential benefits of the study (i.e., either direct benefits to participants or socially beneficial knowledge) outweigh the risks to subjects.

*Data protection and privacy*: Breaches of privacy and confidentiality pose risks to participants. In this scenario, the location data the professor proposes to collect may intrude on her students’ privacy and, if breached, reveal sensitive information like home addresses. When creating a study plan, researchers should consider the least-intrusive means of achieving their research aims and propose clear methods of data protection like encrypting data or stripping it of identifiers.

*Informed consent*: Prior to participating in a study, prospective participants must understand the purpose of the study as well as attendant risks and benefits so they can make an informed decision about participation. This is usually achieved through an “informed consent” process in which the researcher shares key information about a study with a prospective subject, often orally and in writing. Independent review committees often make templates or standard language available to researchers to ensure the disclosure of all necessary information, some of which may be dictated by research regulations.

It is important that consent is not only informed but voluntary, and independent review committees are charged with assuring that consent is obtained under circumstances that minimize the possibility of coercion or undue influence. (Coercion occurs when there is a threat to make an individual worse off or to deny them of something to which they are entitled, and undue influence occurs when something of extreme value is offered to individuals, leading them to make an unreasonable decision)^10^. In the above scenario, committee members may be reasonably concerned that the professor’s students will feel compelled to participate given the power imbalance in their student–teacher relationship. For example, students might worry that they will be graded more harshly if they do not enroll. When developing a study protocol, researchers should take care to consider and address potential threats to voluntariness.

*Incidental findings*: Previously unknown medical conditions may be unintentionally discovered in the course of research; such a discovery is known as an “incidental finding”^11^. In this scenario, it is possible that the professor might detect a heart arrythmia in one of her students that would require medical attention. Researchers should anticipate that incidental findings may arise in their studies and prospectively develop plans for managing them; this might include informing individuals of such findings or referring them to an appropriate clinician.

**Scenario 2:** *An engineering Ph.D. student is developing a t-shirt with an integrated electrothermal heater that can be worn during cold-weather sports activities to improve athletic performance. He has comprehensively tested mechanical robustness and uniform temperatures in the lab and would like to commence testing the t-shirt on people. To avoid having to find volunteers, he plans to test on the shirt on himself and submit the results for publication in an academic journal*.

*Self-experimentation*: Independent review may seem unnecessary when the researcher and human subject are one and the same because we think autonomous individuals can permissibly choose to impose risks and burdens on themselves, but many academic institutions have policies requiring researchers to obtain approval prior to experimenting on themselves^12^. Further, given that the Ph.D. student plans to submit his research for publication, he will likely need evidence of independent ethics review even if the source of his data is self-experimentation. This scenario illustrates the importance of being familiar with local requirements and considering downstream uses of the data to ensure compliance with independent review obligations.

*Scientific rigor*: Independent review committees will often assess whether the proposed research methods are valid and feasible and will answer the research question. In this scenario, the committee will likely worry that having only one research participant means that there will not be sufficient power for data analysis. Because research involves exposing subjects to risks, researchers should design their studies to have clear scientific objectives and utilize accepted methods.

## Additional ethical considerations

Several additional ethical issues fall outside independent review committees’ typical purview review but are worth mentioning. First, many wearable devices that utilize biomedical sensors—such as pulse oximeters and heart rate monitors—have been shown to be less accurate for individuals with darker skin tones^13^. Researchers should take care not to test their prototypes on homogenous groups of individuals, which may introduce bias and limit generalizability.

Second, while there is a tendency to assume that more data is always beneficial, this is not necessarily the case. Consider wearable electroencephalogram (EEG) devices, which are intended to enable better at-home seizure detection and monitoring for individuals with epilepsy. While these devices are being developed with the intention of improving patients’ lives, there might be unintended negative consequences. For example, EEG devices may capture data regarding subclinical seizures (i.e., only detectable via EEG) that could limit individuals’ driving privileges^14^.

Finally, while researchers tend to assume that their wearable devices will be used in the manner intended, others may utilize devices in unexpected ways. In the realm of noninvasive brain stimulation, for instance, early prototypes of simple brain stimulation devices led to the rise of a do-it-yourself community wherein members of the public turned to investigator-published literature to inform their practices^15^, despite warnings that safety was not yet well established. Thus, even during development, researchers should be aware of how their devices and research findings may be used in unintended ways.

## Conclusion

As researchers develop novel wearable health devices and begin testing them with people, it is crucial that they understand and comply with relevant research ethics requirements and regulations. This includes satisfying independent review requirements imposed by their institution, funder, government, or other stakeholders prior to commencing research, as ethics approval cannot be obtained retroactively. For researchers conducting research outside of institutions that have standing review committees, there are independent committees that will review protocols for a fee. Researchers can reach out to the committee reviewing their protocols to ask questions (e.g., about the level of review) and to obtain helpful resources, such as directions for protocol preparation or informed consent form templates. Furthermore, maintaining an awareness of additional ethical considerations—such as the possibility of bias, downstream implications, and unintended uses—can help researchers develop wearable health devices in a responsible manner.
